# Optimizing oocyte yield utilizing a machine learning model for dose and trigger decisions, a multi-center, prospective study

**DOI:** 10.1038/s41598-024-69165-1

**Published:** 2024-08-20

**Authors:** Chelsea Canon, Lily Leibner, Michael Fanton, Zeyu Chang, Vaishali Suraj, Joseph A. Lee, Kevin Loewke, David Hoffman

**Affiliations:** 1 RMA of New York, 635 Madison Avenue, 10th Floor, New York, NY 10022 USA; 2 Alife Health, Inc., 3717 Buchanan Street, Suite 400, San Francisco, CA 94123 USA; 3 IVF Florida, 3251 N State Rd 7 Suite 200, Margate, FL 33063 USA

**Keywords:** Artificial intelligence, Machine learning, Embryo selection, Clinical study, Software, Infertility

## Abstract

The objective of this study was to evaluate clinical outcomes for patients undergoing IVF treatment where an artificial intelligence (AI) platform was utilized by clinicians to help determine the optimal starting dose of FSH and timing of trigger injection. This was a prospective clinical trial with historical control arm. Four physicians from two assisted reproductive technology treatment centers in the United States participated in the study. The treatment arm included patients undergoing autologous IVF cycles between December 2022–April 2023 where the physician use AI to help select starting dose of follicle stimulating hormone (FSH) and trigger injection timing (N = 291). The control arm included historical patients treated where the same doctor did not use AI between September 2021 and September 2022. The main outcome measures were total FSH used and average number of mature metaphase II (MII) oocytes. There was a non-significant trend towards improved patient outcomes and a reduction in FSH with physician use of AI. Overall, the average number of MIIs in the treatment vs. control arm was 12.20 vs 11.24 (improvement = 0.96, p = 0.16). The average number of oocytes retrieved in the treatment vs. control arm was 16.01 vs 14.54 (improvement = 1.47, p = 0.08). The average total FSH in the treatment arm was 3671.95 IUs and the average in the control arm was 3846.29 IUs (difference = −174.35 IUs, p = 0.13). These results suggests that AI can safely assist in refining the starting dose of FSH while narrowing down the timing of the trigger injection during ovarian stimulation, benefiting the patient in optimizing the count of MII oocytes retrieved.

## Introduction

The first step of in-vitro fertilization (IVF) is ovarian stimulation, during which the ovaries are stimulated with gonadotropins to promote follicular growth with the goal of retrieving multiple mature oocytes. During this process, physicians make several clinical decisions that are crucial to the outcome of a cycle. Priority takes place on the dosage and duration of gonadotropins used to stimulate follicles in the ovary prior to final trigger injection to induce ovulation. While the goal of a stimulation cycle is to produce a healthy embryo for a future embryo transfer, to ultimately achieve a live birth, a successful stimulation is needed to achieve that goal. Retrieving a higher number of mature oocytes increases the chances of obtaining high quality, chromosomally normal (euploid) blastocysts, increasing the cumulative chance of live birth from future embryo transfers^1^. However, excessive stimulation can also increase the risk of ovarian hyperstimulation syndrome, although current practices have significantly lowered this risk^2–4^. Thus, the goal of many physicians during ovarian stimulation is to safely maximize the number of oocytes retrieved to increase the number of euploid blastocysts. However, in practice, treatment strategies for ovarian stimulation vary significantly between doctors and clinics, as there exists no standardized approach for making these decisions.

Artificial intelligence (AI) algorithms have been developed to support clinical decision making during ovarian stimulation, with the goal of improving patient outcomes and clinic efficiency by optimizing the dosage and timing of medications to standardize and streamline the IVF process^5–7^. Despite the growing popularity of developing AI for IVF, there is a lack of prospective evidence supporting its efficacy in most studies. Recently, interpretable AI algorithms were developed to help optimize the starting dose of follicle stimulating hormone (FSH) using a patient’s baseline characteristics and the timing of the trigger injection using a patient’s follicle growth and hormone levels^8,9^. Together, these models were integrated into the Stim Assist platform, a clinical decision support software which provides physicians with adjunctive information for the prediction of the number of mature (MII) oocytes that may be retrieved from an ovarian stimulation cycle. The software analyzes patient data to make predictions of MII oocytes for different treatment options. It is intended for use prior to the start of an ovarian stimulation cycle to assist with the selection of starting dose of FSH, as well as throughout the stimulation cycle to assist with the decision of when to administer the trigger injection.

The Eva**l**uat**i**on of the Stim Assist C**l**inical Decision Support Software—An Observational Post-Market Stud**y** (LILY) was designed to collect data on patients undergoing IVF treatment where Stim Assist was utilized by clinicians during stimulation. This study was the first clinical trial to prospectively evaluate the efficacy of AI for optimizing the starting dose of FSH and timing of trigger injection. The primary study endpoint was to compare the mean number of mature oocytes (MII) retrieved for patients undergoing IVF treatment with Stim Assist compared to matched controls from historical patients treated by the same doctor during the year prior.

## Materials and methods

### Ethics approval

This study was conducted accordance with the relevant guideline and regulation following the approval of the research protocol by the institutional review board of Western Copernicus Group (WCG) (study # 20225550). Patient information was deidentified before analysis. A waiver for consent was obtained from the institutional review board of Western Copernicus Group.

### AI models

**Figure 1:**
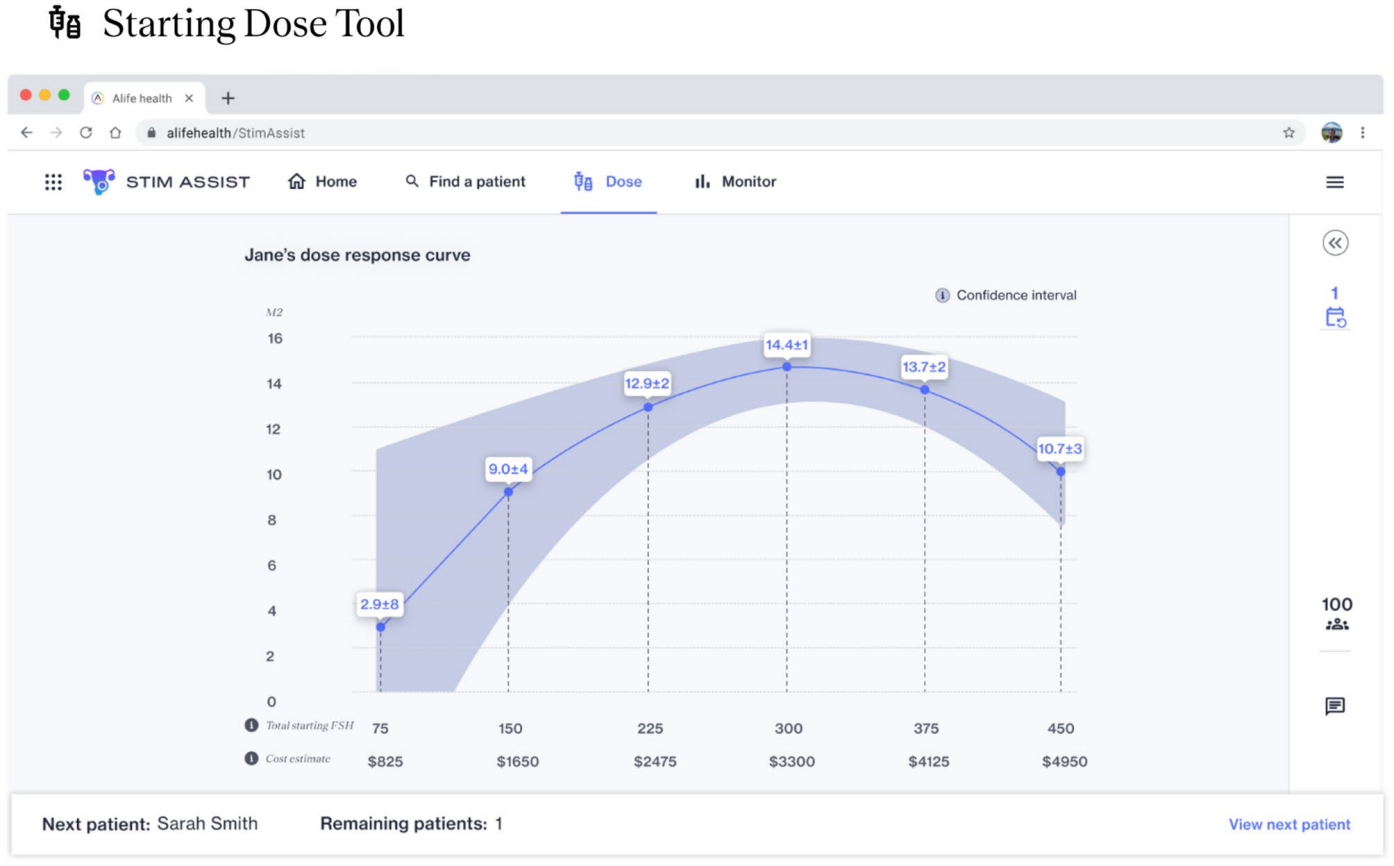
Stim Assist Starting Dose Tool. For a patient of interest, a dose–response curve is created relating the predicted number of MII for different starting doses of FSH. This curve is created using a K-nearest neighbors algorithm to find the 100 most similar patients using patient age, BMI, AMH, and AFC, from a historical dataset containing over 20,000 ovarian stimulation cycles. In this example, the tool predicts that a patient would retrieve the maximum number of MII (14.4 ± 1) at a starting FSH dose of 300 IU.

**Figure 2:**
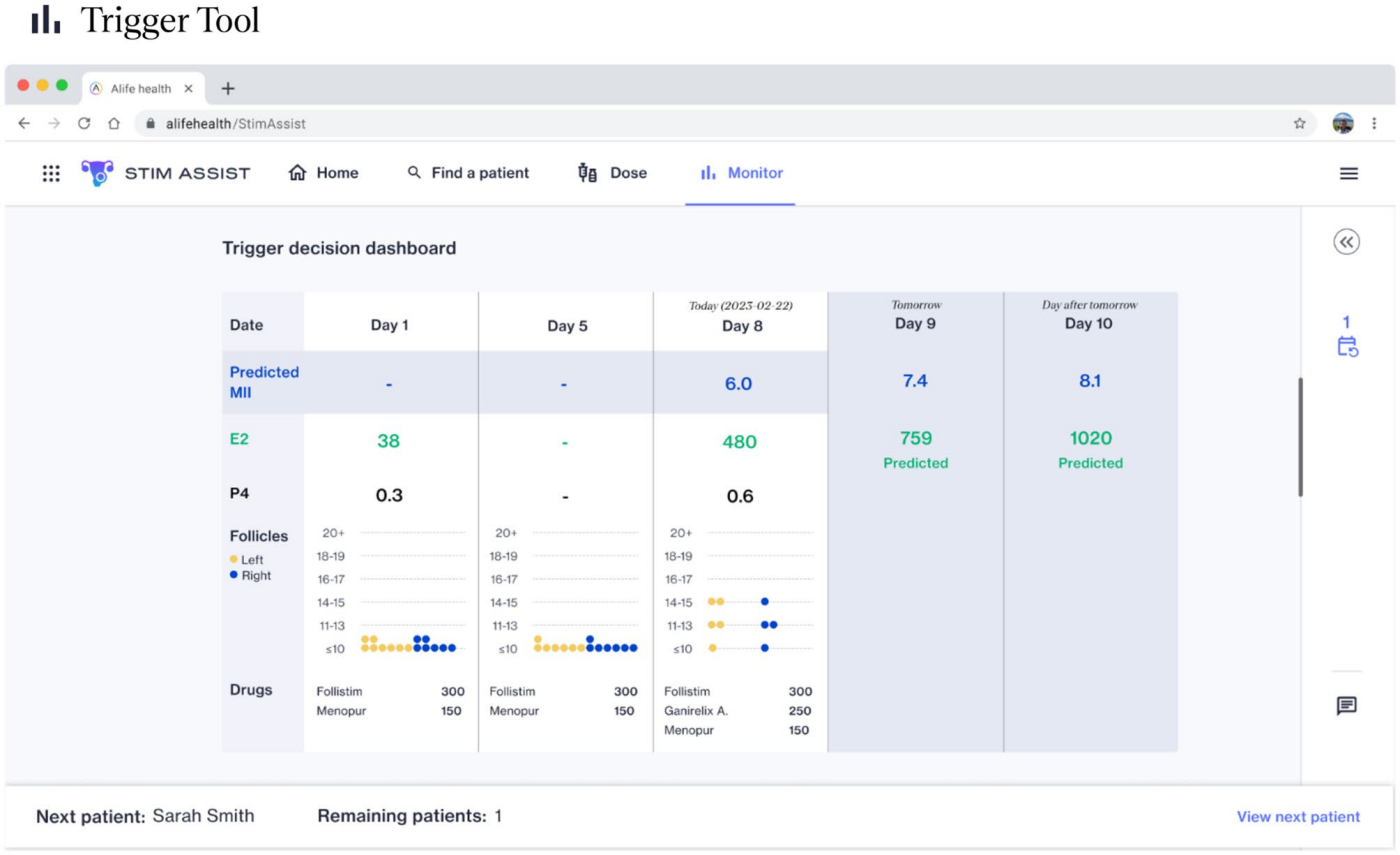
Stim Assist Trigger Tool. During stimulation, the Trigger Tool uses a patient’s E2 and follicles to predict the number of mature eggs if triggering today, tomorrow, or in two days. The patient’s E2 tomorrow and in two days is also predicted. In this example, the Trigger Tool predicts 6.0 MII if triggering today, 7.4 MII if triggering tomorrow, and 8.1 MII if triggering in two days, suggesting that the patient should continue to be stimulated in order to increase egg yield.

Stim Assist is a clinical decision support software intended for use prior to the start of an ovarian stimulation cycle to help optimize the starting dose of FSH and throughout the stimulation cycle to help optimize the timing of the trigger injection. Stim Assist includes two previously published AI algorithms: a Starting Dose Tool (Fig. 1)^8^ and Trigger Tool (Fig. 2)^9^.

The Starting Dose Tool is intended to be used prior to the start of ovarian stimulation to create a dose–response curve to estimate the number of MII eggs that will be retrieved over a range of starting doses of FSH^8^. The algorithm is based on a K-nearest-neighbors model which uses patient age, baseline anti-Mullerian hormone (AMH), baseline antral follicle count (AFC), and body mass index (BMI) to identify 100 most similar patients from a large historical dataset of over 20,000 past patient cycles. Using these 100 similar patients, a dose–response curve (with 95^th^ percentile confidence bands) is generated by fitting a polynomial to the number of MII oocytes relative to the starting dose of FSH across the 100 similar patients. The FSH in this model is calculated as the sum of pure FSH (e.g. Follistim and Gonal-F) plus the FSH component of medication with FSH and LH (e.g. Menopur), measured in international units (IUs).

The Trigger Tool is intended to be used throughout the stimulation cycle to predict the number of MIIs retrieved if triggering today, the number of MIIs retrieved if triggering tomorrow, and the number of MIIs if triggering in two days, as well as the patient’s estradiol (E2) tomorrow^9^. These predictions are made through a set of interpretable linear regression models which take a patient’s current day follicle sizes and E2 level as inputs. To make predictions, the linear regression models take in a patient’s most recent E2 level and follicle measurements, binned into six groups based on their diameters: < 11 mm, 11–13 mm, 16–17 mm, 18–19 mm, and > 19 mm. The Trigger Tool model is designed to only predict biologically feasible trends in MII growth on subsequent days; for example, the model will not predict a decline in MIIs tomorrow, followed by a predicted MII increase in 2 days.

Stim Assist is intended to be used on patients undergoing conventional IVF cycles, and not patients undergoing minimal stimulation IVF or natural cycle IVF. The software is intended for both autologous and donor patient cycles.

### Study design and participants

This was a prospective, observational, post-market study, conducted by four physicians at two clinics in the United States. The treatment arm included 291 patients undergoing IVF treatment where Stim Assist was utilized between December 2022 and April 2023, and the control arm included matched historical patients treated by the same physicians from September 1, 2021 to September 1, 2022. All patients undergoing conventional autologous IVF cycles during the study period were included. None of the data from the LILY study were used for training or testing the AI models.

During the study period, for patients who were treated with the utilization of Stim Assist, the clinician used the Starting Dose Tool, prior to prescribing the initial dose of FSH, to create an individualized dose–response curve to visualize the trade-off between different starting doses of FSH versus predicted MII outcomes. After seeing the dose-response curve, clinicians were allowed to choose any dose they deemed appropriate for the patient. Dose adjustments during stimulation were also allowed, similar to any adjustments the clinician would make without the tool. During each patient monitoring appointment on cycle day 7 or onwards, the clinician used the Trigger Tool to predict the number of MIIs retrieved if triggering today, tomorrow, or in two days, as well as E2 projections if waiting an additional day to trigger. After seeing these predictions, clinicians were allowed to choose whether to continue to push the patient or to administer the trigger injection. Oocyte maturation was induced using recombinant human chorionic gonadotropin (hCG) alone. For patients deemed at risk of ovarian hyperstimulation, oocyte maturation was induced with 4 mg of Lupron or 4 mg of Lupron and 2500 IU hCG at Clinic 1, and 2 mg of Lupron and 1000 IU hCG at Clinic 2^10^.

During the treatment period, each time a patient was viewed in either the Starting Dose Tool or the Trigger Tool, the clinician was instructed to record via a survey whether or not the prediction confirmed or changed their decision, or whether they ignored the prediction due to disagreement or other clinical factors to consider. It was left up to the interpretation of the clinician whether or not their treatment decision was “disagreeing” with the tools, given that the tools did not explicitly make a recommendation, but rather just provided MII predictions. During the control period, patients were treated without the use of Stim Assist, and clinicians made dosing and triggering decisions without being provided AI predictions.

### Data management

Data obtained for this study for the treatment arm were collected directly from the patients’ medical charts and lab notes. At the first clinic, the data were entered directly into Stim Assist. At the second clinic, Stim Assist had direct integration with the clinic’s EMR and thus patient data was automatically transferred to Stim Assist as it was entered into the EMR. Data obtained for the control arm were supplied by the site as de-identified spreadsheets that had been exported from the EMR. The data collected included the primary endpoints of MII oocytes and the secondary endpoint of number of oocytes. Other study variables recorded included patient age, BMI, AMH, AFC, cycle length, daily follicle sizes, daily medications used (e.g. FSH, LH, and trigger injections).

### Statistical analysis

Matching of patients in the treatment-arm to the control-arm was performed for each physician individually, comparing their own treatment-arm patients to their own historical control-arm patients, and matched 1-to-1 based on age, baseline AMH, and baseline AFC. Patients in the treatment-arm with missing baseline AMH and AFC had these values imputed using a K-Nearest Neighbors (KNN) imputer trained on 23,000 prior IVF patients from the Starting Dose Tool dataset^8^. Prior to matching, baseline AMH and AFC were log transformed, and all variables were standardized by subtracting out the mean and dividing by the standard deviation across all patients. Matching was performed, with replacement, using a KNN to find the control-arm patient with the most similar baseline characteristics to each treatment-arm patient. Average outcomes between the matched groups were compared. A t-test was used to determine whether the averages between the two groups were statistically significant.

Additionally, a sub-analysis was performed to approximate the extent to which treatment-arm patients were triggered in accordance with predictions from the Trigger Tool. The inclusion for this sub-analysis was based on criteria that served as proxies for adherence to the tool’s recommendations: (1) on the day of trigger, the model did not predict an increase in MIIs if waiting to trigger tomorrow, and (2) on the previous visit before trigger day, the model predicted at least a 5% increase in MIIs if waiting to trigger tomorrow. Patients were also included if they had E2 > 5000 pg/mL on the day of trigger, indicating a conservative threshold to mitigate the risk of ovarian hyperstimulation syndrome (OHSS)^11^. These criteria were chosen to identify which patients were stimulated until our model predicted a decline in MIIs or until their E2 level reached this threshold. It is important to note that these criteria represent an approximate method of assessing whether the actual clinical decisions to trigger are aligned with the model's predictions, as the true reasons behind each triggering decision can be complex and vary based on protocol. Treatment-arm patients who were triggered in accordance with the Trigger Tool were matched to control-arm patients using the same methodology as above.

A final sub-analysis was performed to understand whether dosing and outcomes differed between patients where the clinician agreed with Stim Assist predictions versus patients where the clinician disagreed, as recorded by the survey. Patients in the treatment arm were separated into two groups: a first group of patients where all of the Stim Assist predictions either confirmed or changed the clinician’s decision, and a second group of patients where any of the Stim Assist predictions were ignored due to disagreement or other clinical factors to consider. Patients in the “agree” and “disagree” groups were matched to control-arm patients using the same matching methodology, and the changes in outcomes between the two groups were compared.

## Results

There were 291 patients who underwent IVF treatment where Stim Assist was used by their physician. Patient characteristics are summarized in Table 1. In the treatment group, one patient had AMH imputed, 16 patients had AFC imputed, and one patient had BMI imputed. After propensity matching, there were no significant differences in age, AMH, AFC, or BMI between treatment and control arms.

**Table 1:** Patient characteristics for treatment-arm and control-arm.

Demographic	Treatment arm	Control arm
N	291	291
Age (years)		
All	36.10	36.07
Physician 1	36.48	36.38
Physician 2	35.54	35.45
Physician 3	35.94	35.94
Physician 4	36.91	37.18
AMH (ng/mL)		
All	2.63	2.50
Physician 1	3.45	3.03
Physician 2	2.45	2.55
Physician 3	2.01	1.99
Physician 4	2.06	1.85
AFC (number)		
All	17.20	16.47
Physician 1	17.79	16.23
Physician 2	17.86	17.52
Physician 3	16.04	15.67
Physician 4	15.88	15.69
BMI (kg/m^2^)		
All	24.66	25.68
Physician 1	26.54	26.39
Physician 2	23.47	25.22
Physician 3	23.75	25.98
Physician 4	24.51	24.53

Average laboratory outcomes between the treatment-arm and matched control-arm are summarized in Table 2. Overall, the average number of MIIs in the treatment vs. control arm was 12.20 vs 11.24 (improvement = 0.96, p = 0.16). When analyzed per physician, the average number of MIIs in treatment vs. control arm was 10.62 vs 9.52 for physician 1 (improvement = 1.11, N = 95, p = 0.25), 13.29 vs. 12.94 for physician 2 (improvement = 0.35, N = 97, p = 0.77), 11.67 vs. 11.19 for physician 3 (improvement = 0.48, N = 63, p = 0.75), and 14.28 vs. 11.28 for physician 4 (improvement = 3.0, N = 36, p = 0.21). The average number of oocytes retrieved in the treatment vs. control arm was 16.01 vs 14.54 (improvement = 1.47, p = 0.08). When analyzed per physician, the average number of oocytes in treatment vs. control arm was 14.07 vs 12.48 for physician 1 (improvement = 1.59, N = 95, p = 0.24), 17.7 vs. 16.33 for physician 2 (improvement = 1.37, N = 97, p = 0.36), 15.3 vs. 14.65 for physician 3 (improvement = 0.65, N = 63, p = 0.71), and 17.78 vs. 14.92 for physician 4 (improvement = 2.86, N = 36, p = 0.31).

**Table 2:** Outcomes between treatment-arm and matched control-arm.

Physicians	N	Eggs retrieved			MII retrieved			Total FSH		
		Treatment	Control	Delta	Treatment	Control	Delta	Treatment	Control	Delta
All	291	16.01	14.54	1.47	12.20	11.24	0.96	3671.95	3846.29	−174.35
Physician 1	95	14.07	12.48	1.59	10.62	9.52	1.11	2803.08	3331.06	−527.98
Physician 2	97	17.70	16.33	1.37	13.29	12.94	0.35	3955.73	4024.74	−69.01
Physician 3	63	15.30	14.65	0.65	11.67	11.19	0.48	3977.38	4040.08	−62.70
Physician 4	36	17.78	14.92	2.86	14.28	11.28	3.00	4702.14	4401.43	300.71

The average total FSH in the treatment arm was 3671.95 IUs and the average in the control arm was 3846.29 IUs (difference = -174.35 IUs, p = 0.13). The average day of trigger was 10.27 in the treatment group and 10.00 in the control group (difference =  + 0.27, p = 0.07).

For patients triggered in accordance with the Trigger Tool predictions (Table 3), the overall treatment vs. control arm MIIs were 15.22 vs. 12.65 (improvement = 2.57, N = 97, p = 0.02), and when analyzed per physician, were 14.72 vs 11.97 (improvement = 2.76, N = 29, p = 0.09), 15.91 vs. 13.41 (improvement = 2.5, N = 34, p = 0.22), 15.0 vs. 12.74 (improvement = 2.26, N = 23, p = 0.27) and 14.82 vs. 11.91 (improvement = 2.91, N = 11, p = 0.49).

**Table 3:** Outcomes between treatment-arm and matched control-arm for patients who were triggered in accordance with Trigger Tool predictions.

Physicians	N	Eggs retrieved			MII retrieved			Total FSH		
		Treatment	Control	Delta	Treatment	Control	Delta	Treatment	Control	Delta
All	97	19.73	16.47	3.26	15.22	12.65	2.57	3481.37	3569.67	−88.31
Physician 1	29	18.93	15.69	3.24	14.72	11.97	2.76	2864.40	2990.98	−126.59
Physician 2	34	21.21	17.03	4.18	15.91	13.41	2.50	3738.24	3685.29	52.94
Physician 3	23	19.35	17.57	1.78	15.00	12.74	2.26	3351.09	3713.04	−361.96
Physician 4	11	18.09	14.55	3.55	14.82	11.91	2.91	4586.36	4525.00	61.36

For the Starting Dose Tool, clinicians reported that the predictions confirmed or changed their decisions 63.1% of the time. They disagreed with the predictions 32.4% of the time, and in 4.5% of cases, predictions were unavailable due to missing data. For the Trigger Tool, the predictions either confirmed or changed clinicians' decisions 78.4% of the time, with disagreements occurring 17.0% of the time. Predictions were unavailable 4.6% of the time due to missing data.

Treatment-arm patients were separated into Stim Assist “agree” and “disagree” groups. In 55.7% of cycles, clinicians “agreed” (i.e. confirmed or changed their decision) with every Stim Assist prediction shown to them during that cycle. In the remaining 44.3% of cycles, clinicians “disagreed” (i.e. ignored due to disagreement or other clinical factors to consider) with at least one of the Stim Assist predictions shown to them during that cycle. In the Stim Assist agree group, the overall treatment vs. control arm MIIs were 10.99 vs. 9.96 (improvement = 1.03, p = 0.22), and the overall treatment vs. control arm total FSH was 3317.24 vs 3647.30 (difference = −330.06, p = 0.09). In the Stim Assist disagree group, the overall treatment vs. control arm MIIs were 12.64 vs. 11.82 (improvement = 0.82, p = 0.47), and the overall treatment vs. control arm total FSH was 3651.56 vs 3969.94 (difference = −318.37, p = 0.11).

The use of Stim Assist did not introduce any adverse events. There were no reported cases of ovarian hyperstimulation syndrome in either the treatment or control group.

## Discussion

To our knowledge, this clinical study is the first to evaluate the efficacy of AI for optimizing ovarian stimulation using prospective post-market data. In this comparative study, Stim Assist was used to confirm or change the decision of starting dose or trigger timing for the majority of patients in the treatment arm. Although not statistically significant, this resulted in an increase in MIIs by 0.96 for the treatment arm.

Stim Assist demonstrates the potential to maximize laboratory outcomes by providing personalized data driven recommendations for starting dose of FSH and day of trigger. A previous study on the Starting Dose Tool retrospectively estimated that outcomes could be improved by up to 1.5 MIIs for certain patients, while others could save up to 1400 IUs of medication^8^. Similarly, it was retrospectively estimated that the Trigger Tool could improve outcomes by up to 2.7 MIIs^9^. The present study is the first to prospectively evaluate the impact of technologies where clinicians used the tools to help make or confirm decisions. Although the observed increase in MIIs with the use of the tool was not statistically significant due to an insufficient sample size, the trend towards improved outcomes was aligned with predicted improvements in prior work.

Patients were stimulated slightly longer when clinicians used Stim Assist, possibly explaining the trend towards improved outcomes in the treatment arm. On average, patients in the treatment group were stimulated for 0.26 days longer than patients in the matched control group, while there was a slight reduction in the total amount of FSH used to stimulate the patient. Furthermore, we found that the proportion of patients who were treated in accordance with Trigger Tool predictions increased by approximately 3.2% with the use of Stim Assist. Patients who were treated in accordance with the Trigger Tool showed a greater improvement in MIIs retrieved. These incremental changes in treatment patterns could suggest that the adjunctive information provided to clinicians by Stim Assist could have helped to optimize outcomes for some patients.

During this study, Stim Assist was utilized as an adjunctive tool in accordance with its intended commercial use. AI predictions were shown to the clinicians to help guide or confirm their decisions on dosing and trigger injection timing. The Starting Dose Tool was intended to help clinicians better understand the trade-off between FSH dosing and MII yield. The MII and E2 predictions from the Trigger Tool were intended to help clinicians understand how to adjust the stimulation duration to safely maximize MIIs. However, all treatment decisions during the study period were ultimately left to the clinician. Future studies should explore using Stim Assist prescriptively to understand how medication dosing, patient complications (specifically OHSS), and patient outcomes would change if treatment decisions were set directly by AI tools only.

The survey results indicated that Stim Assist either confirmed or changed clinicians’ decisions the majority of the time. We compared patients where clinicians “agreed” with all Stim Assist predictions to patients where clinicians “disagreed” with at least one prediction during their cycle. When comparing the treatment arm to matched historical controls, treatment arm patients where clinicians agreed had a slightly greater improvement in outcomes and larger reduction in total FSH compared to patients where clinicians noted disagreement at least once. Interestingly, the “disagree” patients had a higher number of mature eggs retrieved on average compared to the “agree” patients, indicating that these patients tended to be higher responders. For many of these higher responders, it is likely that the Trigger Tool predicted increased MIIs if continuing stimulation even late into the cycle, whereas the clinician decided to administer the trigger injection earlier to avoid hyperstimulation risk or because the patient was predicted to have sufficient outcomes.

Ultimately, Stim Assist represents a data-driven approach to help standardize treatment decisions of starting FSH dose and trigger timing, which currently can vary significantly between doctors and clinics. For example, patients are generally dosed with FSH based on their age and ovarian reserve, but this choice is highly dependent on a clinician’s training and personal experiences. The relationship between FSH dose and ovarian response is complex; dose response studies have been limited in sample size^12,13^, whereas larger retrospective studies have shown conflicting evidence on whether outcomes improve with higher doses of medication^14,15^. The Starting Dose Tool provides an objective and personalized measurement of how a patient is likely to respond at different doses of FSH using historical data from patients with similar baseline measurements. Likewise, for determining timing of the trigger injection, the standard practice of monitoring the size of the leading follicles often varies between practices^16^. The Trigger Tool provides higher fidelity than the current practice of lead follicle monitoring by taking into account all measured follicles to make a prediction of how many MIIs would be retrieved if the patient was triggered or stimulation was continued^9^. By standardizing treatment decisions, this tool also has the potential to reduce the cognitive load on clinicians, who sometimes have to make hundreds of stimulation treatment decisions per day, to help confidently make and confirm these decisions more efficiently.

The promising trend towards improved laboratory outcomes with use of Stim Assist suggests that this tool could be evaluated in the future in its ability to enable non-specialized clinicians or advanced practice providers to manage or monitor more straightforward stimulation cases, under the supervision of an infertility physician. This could allow for more highly trained reproductive endocrinologists to focus more time on complex clinical cases. Another potential application of this technology is for training purposes. The Starting Dose and Trigger Tools are directly interpretable, meaning the basis for their predictions can be clearly explained, which could further help with training as well as general clinical trust and adoption^17^. Future clinical studies should evaluate the capability of this tool to assist younger clinicians, such as residents or fellows, in making dosing and trigger decisions.

MIIs were chosen as the primary outcome in this study because the number of mature oocytes is a direct outcome of ovarian stimulation. Furthermore, the Stim Assist tools have been designed specifically to maximize the number of MIIs^8,9^. Post-fertilization outcomes, such as 2PNs, euploid blastocysts, or birth rates, also depend on the quality of the sperm and laboratory procedures^18^, which Stim Assist is currently not designed to help optimize. Furthermore, using MIIs as the primary outcome allowed for the inclusion of egg freezing patients in our study protocol. However, the ultimate goal of IVF is the birth of a healthy baby. Retrieving more MIIs increases chances of euploid blastocysts post-fertilization, which ultimately increase chances of live birth. The positive associations between increased egg yield resulting in increased 2PNs, blastocysts, and live birth rates has been established in numerous prior studies^1,19–21^, but future clinical studies should directly analyze whether use of Stim Assist can measurably increase more pertinent outcomes, such as euploid rate, pregnancy rates, and live birth rates, after implementation at a clinic.

The study has multiple strengths, including the prospective design with a rigorous propensity matched control arm for comparisons. Few studies of AI in IVF thus far have addressed safety and efficacy of their models within a prospective clinical setting^5^. However, the study is not without limitations, which include that this study was not randomized by design. The study was also only conducted at two clinics with four physicians, with a limited patient sample size which resulted in the change in outcomes not reaching statistical significance. Given the novel application of AI within the clinical setting of ovarian stimulation, this initial study was designed to be smaller in scale to establish initial findings, which will ultimately inform how to power larger future prospective studies using these tools. Furthermore, to generalize these results to a larger population, future prospective studies are needed to analyze multiple sites with many different physicians, as each physician may interpret Stim Assist predictions differently based on their experience. Additionally, Stim Assist includes both the Starting Dose Tool and the Trigger Tool algorithms, meaning that it was not possible to differentiate the impact of each individual algorithm on patient outcomes. Given that this was a post-market study, the goal of this initial study was to evaluate the effect of clinicians using the full Stim Assist software, but future studies should isolate each algorithm to better understand the individual effects of the Starting Dose Tool and Trigger Tool.

## Conclusion

This clinical study is the first to prospectively investigate the use of AI for optimizing starting dose of FSH and timing of the trigger injection, through the use of Stim Assist. Each doctor included in the study showed a promising trend towards improving patient laboratory outcomes with the use of Stim Assist. The use of Stim Assist did not introduce any adverse events. Together, these results provide the first prospective evidence that AI can safely and effectively be used to help optimize ovarian stimulation.

## Data Availability

Data Availability The data that supports the findings of this study is available from the corresponding author upon reasonable request and approvals from Alife Health, RMANY, and IVF Florida.
